# Correction: Global abundance patterns, diversity, and ecology of *Patescibacteria* in wastewater treatment plants

**DOI:** 10.1186/s40168-025-02330-4

**Published:** 2026-01-05

**Authors:** Huifeng Hu, Jannie Munk Kristensen, Craig William Herbold, Petra Pjevac, Katharina Kitzinger, Bela Hausmann, Morten Kam Dahl Dueholm, Per Halkjaer Nielsen, Michael Wagner

**Affiliations:** 1 Centre for Microbiology and Environmental Systems Science, University of Vienna, Djerassiplatz 1, Vienna, 1030 Austria; 2 Center for Microbial Communities, Department of Chemistry and Bioscience, Aalborg University, Aalborg, Denmark; 3 Joint Microbiome Facility of the Medical University of Vienna, University of Vienna, Vienna, Austria; 4 Division of Clinical Microbiology, Department of Laboratory Medicine, Medical University of Vienna, Vienna, Austria; 5 Doctoral School in Microbiology and Environmental Science, University of Vienna, Universitätsring 1, Vienna, 1010 Austria; 6 Te Kura Putaiao Koiora, School of Biological Sciences, Te Whare Wananga o Waitaha, University of Canterbury, Otautahi, Christchurch, Aotearoa, New Zealand

**Correction: Microbiome 12, 55 (2024)**

**https://doi.org/10.1186/s40168-024-01769-1**

Following publication of the original article [1], the author reported that in the Methods section, the PCR conditions were reported as “55 °C for 60 s, 72 °C for 120 s”; however, the correct conditions are “55 °C for 120 s, 72 °C for 60 s.”

The original article has been updated.
