# Supplementary material for: Global abundance patterns, diversity, and ecology of Patescibacteria in wastewater treatment plants
Source: Microbiome. 2024 Mar 16;12:55. doi: 10.1186/s40168-024-01769-1 (PMC10943839; doi:10.1186/s40168-024-01769-1)
Supplement: Supplementary file 3 — Additional file 2: Table S1. Primer sequences used for the in silico coverage analyses. Table S2. Coverages of commonly used 16S rRNA gene primers (V1-V3, V3-V4, V4, V4-V5, and modified V4) for different phyla evaluated by the SILVA r138.1 database with zero and one mismatch. Table S3. Coverages of commonly used 16S rRNA gene primers (V1-V3, V3-V4, V4, V4-V5, and modified V4) for different classes of Patescibacteria evaluated by the SILVA r138.1 database with zero and one mismatch. Table S4. Coverages of commonly used 16S rRNA gene primers (V1-V3, V3-V4, V4, V4-V5, and modified V4) for different phyla evaluated by the MiDAS database 4.8.1 with zero and one mismatch. Table S5. Coverages of commonly used 16S rRNA gene primers (V1-V3, V3-V4, V4, V4-V5, and modified V4) for different classes of Patescibacteria evaluated by the MiDAS database 4.8.1 with zero and one mismatch. Table S6. Primer sequences for the modified V4 primer pair compared with the original V4 primer pair. Table S7. Metadata for wastewater treatment plants. Table S8. Genus level richness comparisons between the data sets obtained by the original and the modified primer pairs. The slope of the observed ASV and Shannon index were predicted by the linear regression analysis using the modified and original primer sets. Table S9. Blast mapping of patescibacterial ASVs to Danish WWTP metagenomic datasets. Three 100% identity mapping with >330 base pair coverage were highlighted in yellow. Table S10. Global distribution of the patescibacterial genera identified as core community members from each country (average relative abundance). Table S11. Core patescibacterial genera identified if all four main process types of activated sludge samples were analysed together and MAG representation of core genera. Table S12. Core patescibacterial genera identified in activated sludge samples from plants with carbon removal (C). Table S13. Core patescibacterial genera identified in activated sludge samples from plants wi [file 40168_2024_1769_MOESM2_ESM.pdf]

# Supplementary Information

## Global abundance patterns, diversity, and ecology of *Patescibacteria* in wastewater treatment plants

Huifeng Hu[1,5], Jannie Munk Kristensen[1,2], Craig William Herbold[1,6], Petra Pjevac[1,3], Katharina Kitzinger[1], Bela Hausmann[3,4], Morten Kam Dahl Dueholm[2], Per Halkjaer Nielsen[2], Michael Wagner[1,2,3]

1. Centre for Microbiology and Environmental Systems Science, University of Vienna. Djerassiplatz 1, 1030 Vienna, Austria
2. Center for Microbial Communities, Department of Chemistry and Bioscience, Aalborg University, Aalborg, Denmark
3. Joint Microbiome Facility of the Medical University of Vienna and the University of Vienna, Vienna, Austria
4. Division of Clinical Microbiology, Department of Laboratory Medicine, Medical University of Vienna, Vienna, Austria
5. Doctoral School in Microbiology and Environmental Science, University of Vienna. Universitätsring 1, 1010 Vienna, Austria
6. Te Kura Pūtaiao Koiora | School of Biological Sciences, Te Whare Wānanga o Waitaha | University of Canterbury, Christchurch, New Zealand

## Supplementary Figures

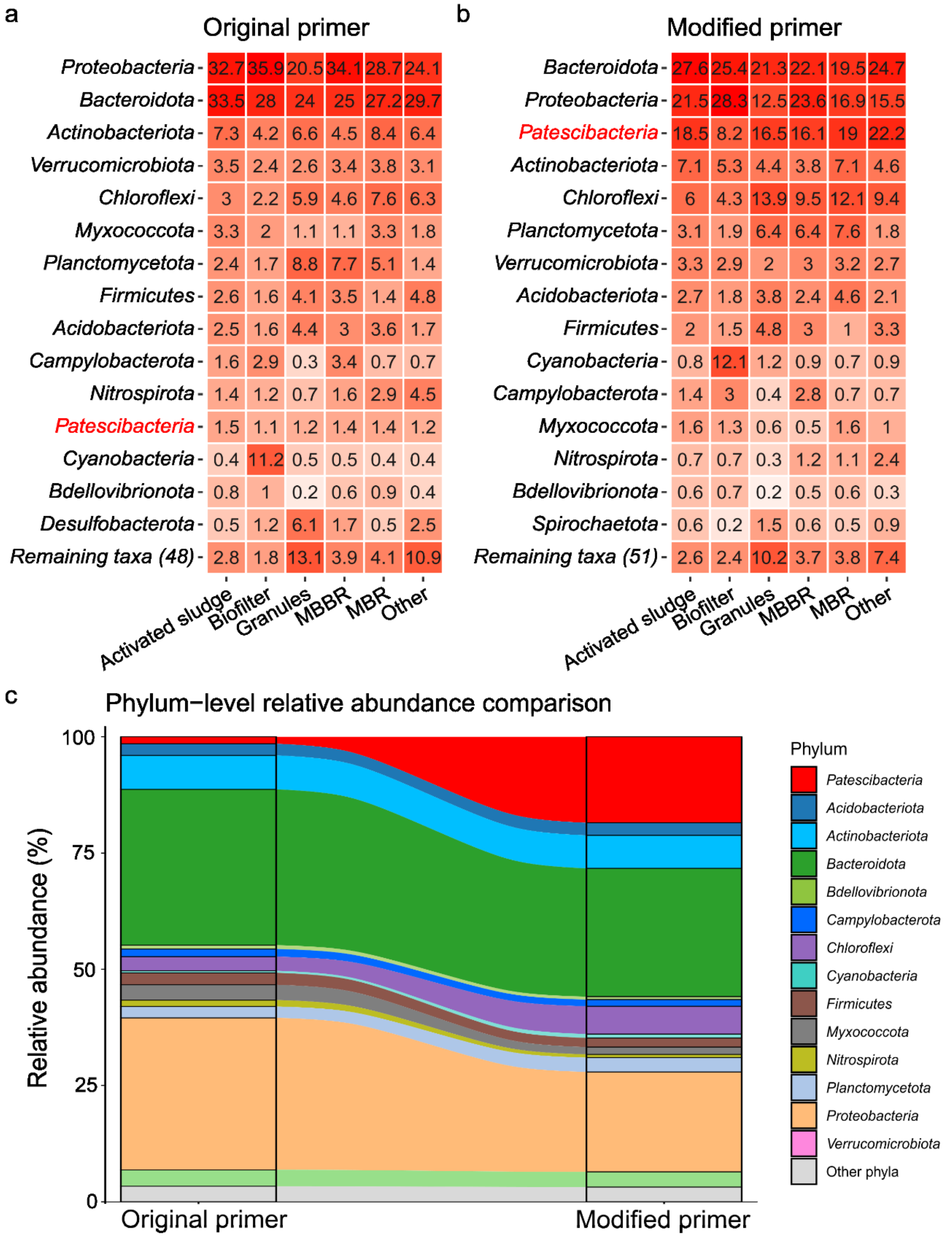


**Supplementary Figure 1. Heatmap of phylum-level abundance grouped by plant type.** Average abundance of top 15 dominant phyla across different plant types revealed by the original primers (a) and the modified primers (b). The heatmap shows average relative abundance in % for each phylum and plant type. The alluvial-bar plot shows changes in relative abundance of top phyla in the activated sludge systems between original and modified primers (c).

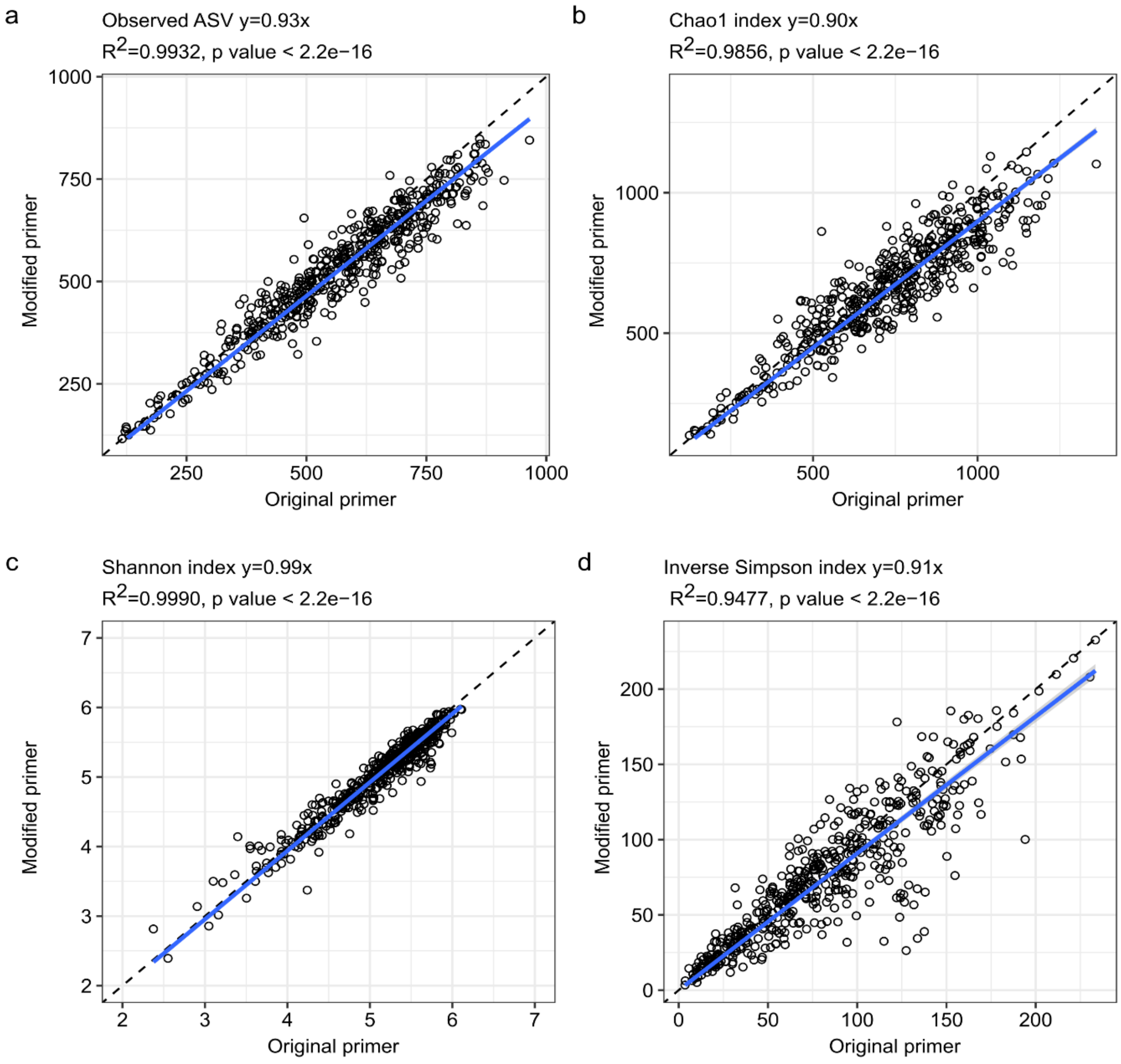


**Supplementary Figure 2. Non-patescibacterial ASV richness comparison between the original and the modified primer pair.** Regression analysis of alpha diversity indices (ASV richness, chao1 index, Shannon index and inverse Simpson index) obtained by the modified primer pair and the original primer pair of each sample after removing reads classified as *Patescibacteria* (a-d). Coefficient, $R^2$ value and *p* value are indicated in the title of each panel. The observed regression line is depicted in blue, the theoretical 1:1 line is indicated as a dashed line.

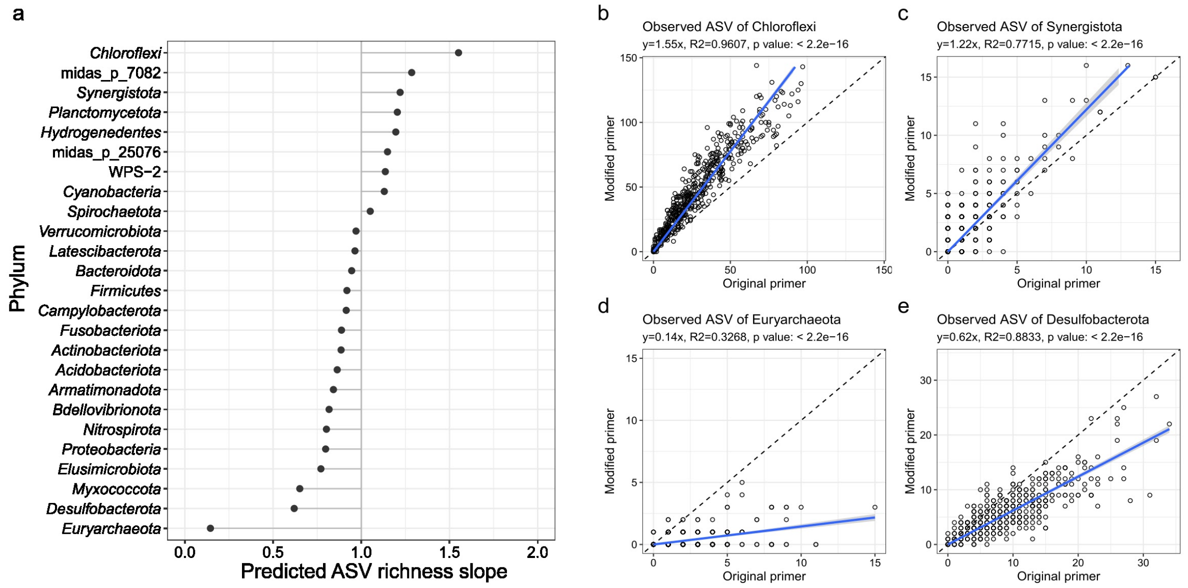


**Supplementary Figure 3. Phylum level richness comparison between the original and the modified primer pair.** Panel a: The lollipop plot shows for different phyla the predicted ASV richness slope by a linear regression analysis between the ASV richness revealed in the overall 16S rRNA gene amplicon data set by the original primer pair and the modified primer pair. A slope of 1 means equal representation of a phylum by both primer pairs, a slope >1 higher representation, and a slope <1 lower representation by the modified primers. Only phyla with >0.1% average relative abundance in activated sludge samples are shown. Panel b-e: Regression analysis of ASV richness of *Chloroflexi*, *Synergistota*, *Euryachaeota* and *Desulfobacterota* obtained by the modified primer pair and the original primer pair of each sample after removing reads classified as *Patescibacteria*. Coefficient, $R^2$ value and p value are indicated in the title of each panel. The observed regression line is depicted in blue, the theoretical 1:1 line is indicated as a dashed line.

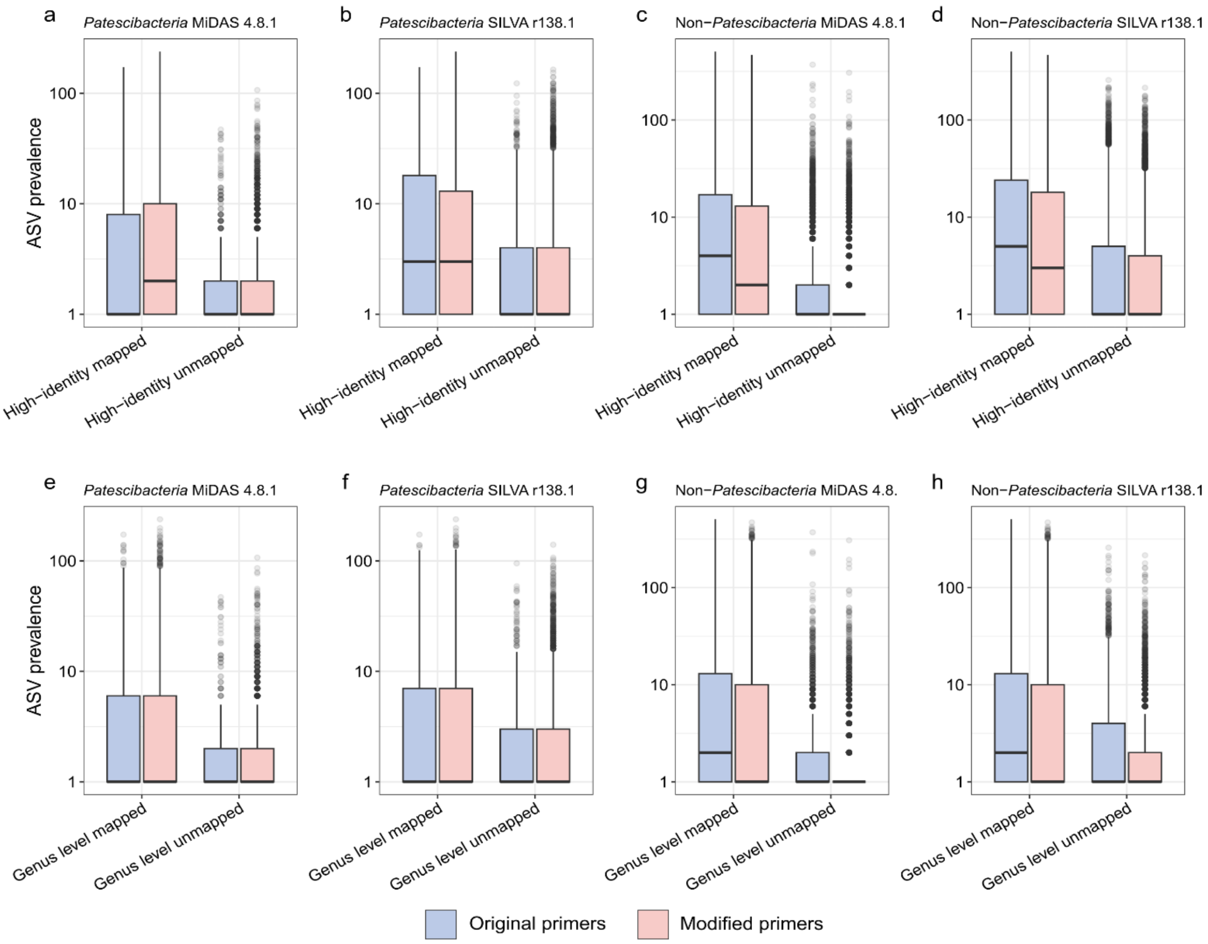


**Supplementary Figure 4. Prevalence of novel patescibacterial and non-patescibacterial ASVs.** Prevalence of patescibacterial and non-patescibacterial ASVs without high identity (99%) hits (a-d) and without genus level identity (94.5%) hits (e-h) against the MiDAS 4.8.1 and the SILVA r138.1 databases. Y-axis indicates the number of samples where each ASV was detected by the original primer pair and the modified primer pair.

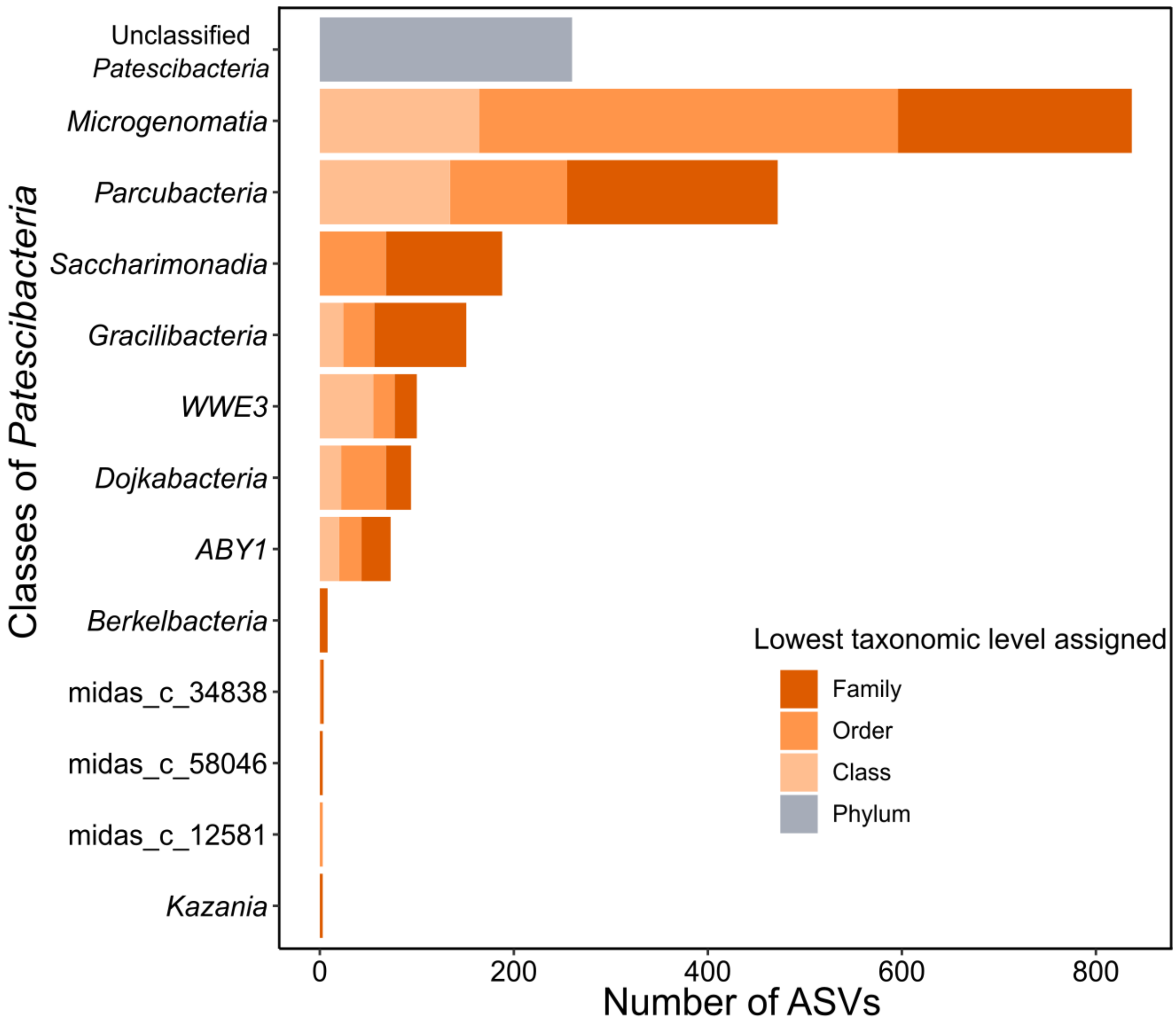


**Supplementary Figure 5. Taxonomic affiliation of unclassified ASVs.** Number of genus level unclassified ASVs in each class of *Patescibacteria* (Y-axis). The stacked barplot colours indicate for each *Patescibacteria* class the number of retrieved ASVs at their annotated taxonomic levels using the assignTaxonomy function in DADA2 against the MiDAS 4.8.1 database.

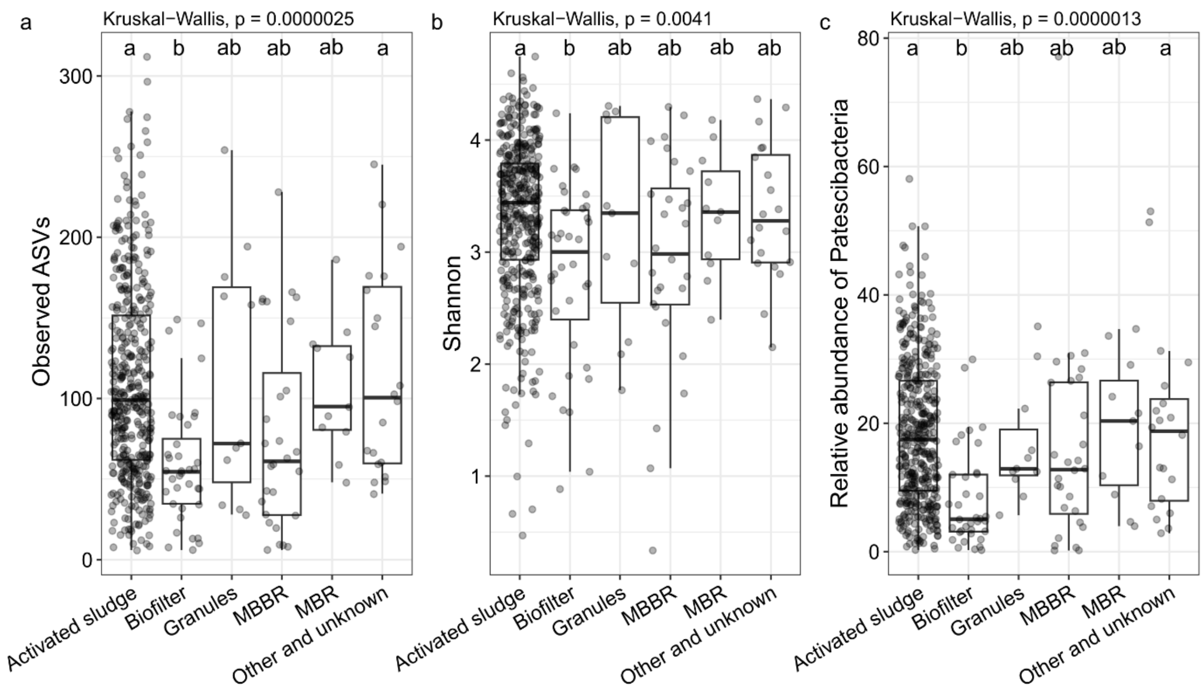


**Supplementary Figure 6. Richness and abundance of *Patescibacteria* in samples of different WWTP types.** Observed number of ASVs (a), Shannon index (b) and relative abundance (c) of *Patescibacteria* in different plant types. The non-parametric Kruskal-Wallis test was used to infer significant differences between plant types. Pairwise comparisons of plant types were done by a post-hoc Dunn's test (Bonferroni correction, α=0.01). The results are shown with compact letter display (groups that do not share letters are significantly different).

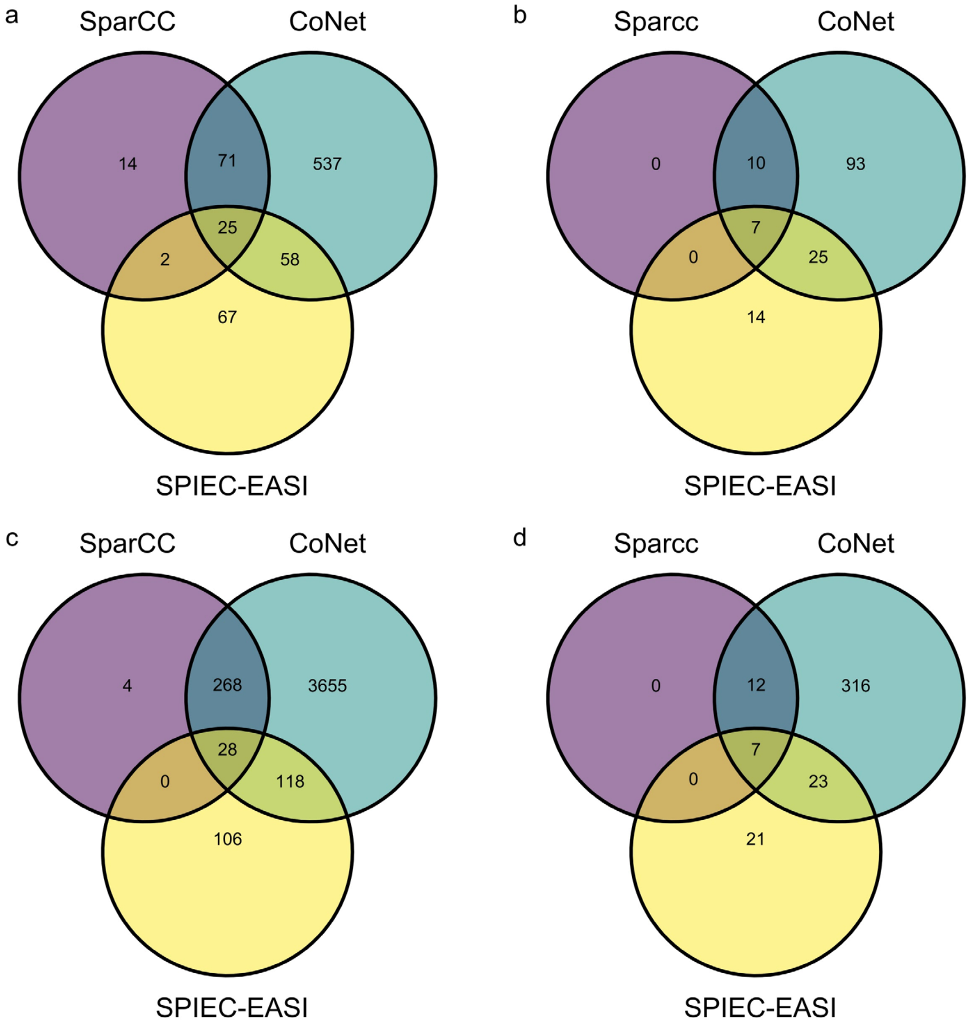


**Supplementary Figure 7. Overlap of edges supported in networks inferred by SparCC, CoNet and SPIEC-EASI.** Panel a: overlap of connections between all taxa at genus level; panel b: overlap of connections to *Patescibacteria* at genus level; panel c: overlap of connections between all taxa at ASV level; panel d: overlap of connections to *Patescibacteria* at ASV level.

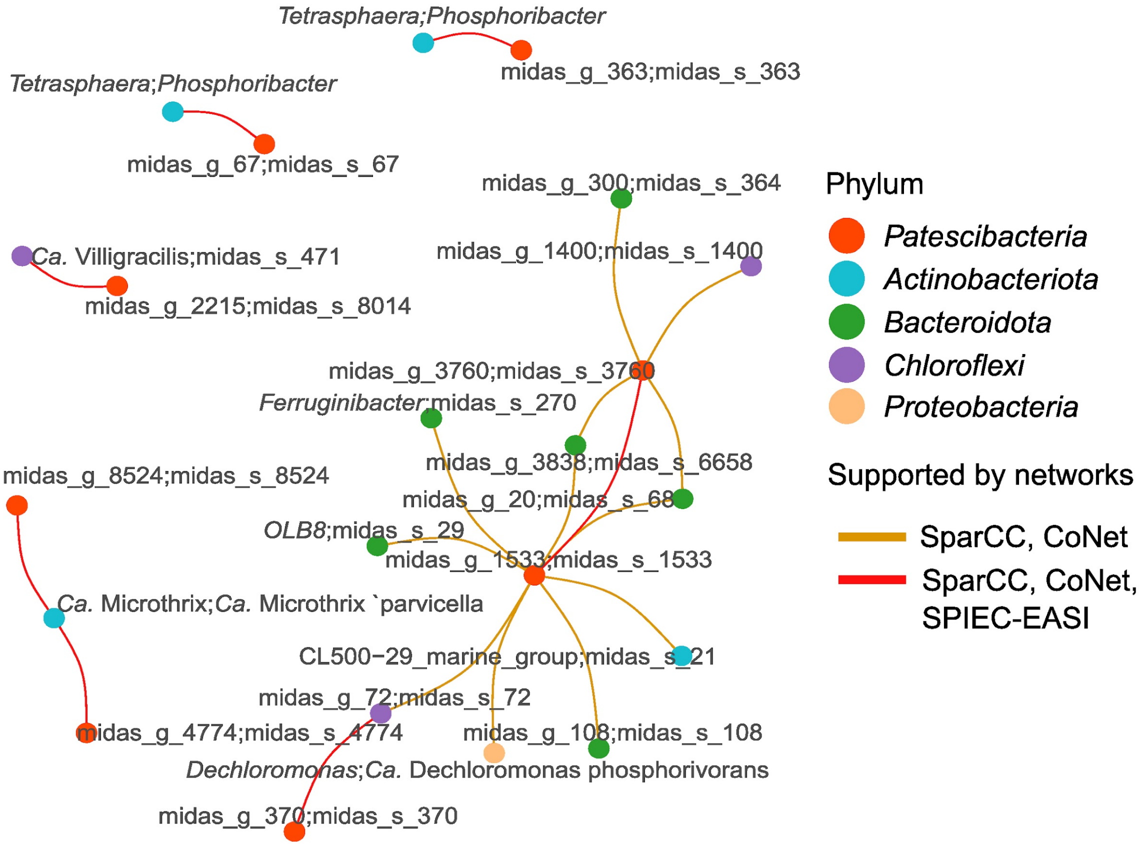


**Supplementary Figure 8. ASV level co-occurrence network reveals potential host-association of *Patescibacteria*.** ASV pairs including patescibacterial ASVs with *p* value <0.001 and absolute correlation value >0.5 are shown in this network. Each node represents an ASV, labelled by the species and genus name. The node color indicates different phyla. Connections between nodes also supported by networks constructed by other methods (CoNet and SPIEC-EASI) are marked by different color.

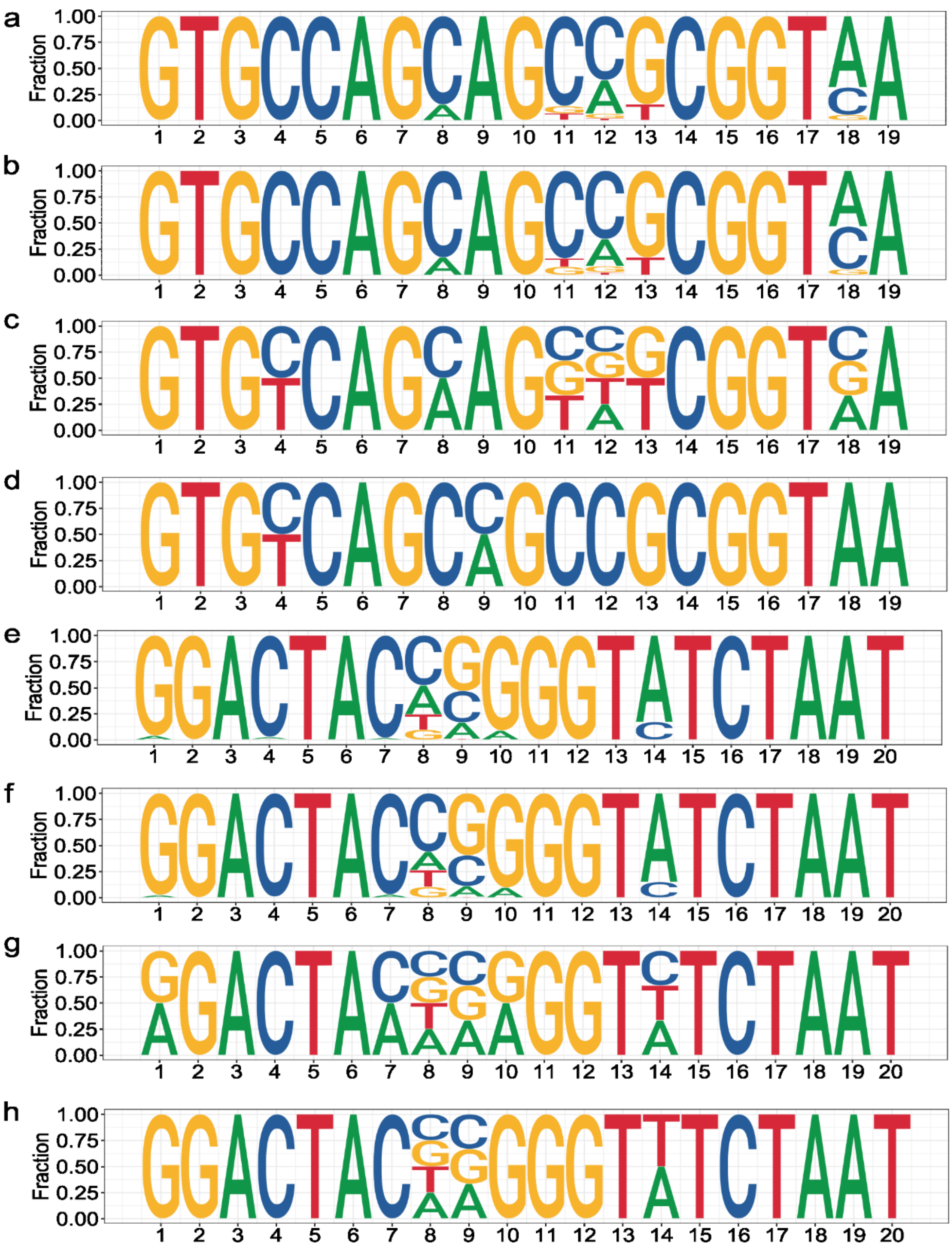


**Supplementary Figure 9. Primer binding region of *Patescibacteria* 16S rRNA gene.** Base composition of the binding sites of *Patescibacteria* 16S rRNA gene sequences for the 515F (positions 515-533; panel a-d)/806R (positions 787-806, panel e-h) primer pair that is widely used for amplicon sequencing. Panels a) and b) show *Patescibacteria* 16S rRNA gene sequences of the forward primer binding site retrieved from the MiDAS (4.8.1) and SILVA database (r138.1), respectively. Panels c) and d) show the modified and original forward primer sequence, respectively. Panels e) and f) show *Patescibacteria* 16S rRNA gene sequences of the reverse primer binding site retrieved from the MiDAS (4.8.1) and SILVA database (r138.1), respectively. Panels g) and h) show the modified and original reverse primer sequence, respectively. The Y-axis represents the fraction of A,C,G and T, X-axis represents the position of bases in the primer sequences.

**Supplementary table legends**

Table S1. Primer sequences used for the *in silico* coverage analyses.

Table S2. Coverages of commonly used 16S rRNA gene primers (V1-V3, V3-V4, V4, V4-V5, and modified V4) for different phyla evaluated by the SILVA r138.1 database with zero and one mismatch.

Table S3. Coverages of commonly used 16S rRNA gene primers (V1-V3, V3-V4, V4, V4-V5, and modified V4) for different classes of *Patescibacteria* evaluated by the SILVA r138.1 database with zero and one mismatch.

Table S4. Coverages of commonly used 16S rRNA gene primers (V1-V3, V3-V4, V4, V4-V5, and modified V4) for different phyla evaluated by the MiDAS 4.8.1 database with zero and one mismatch.

Table S5. Coverages of commonly used 16S rRNA gene primers (V1-V3, V3-V4, V4, V4-V5, and modified V4) for different classes of *Patescibacteria* evaluated by the MiDAS 4.8.1 database with zero and one mismatch.

Table S6. Primer sequences for the modified V4 primer pair compared with the original V4 primer pair

Table S7. Metadata for wastewater treatment plants.

Table S8. Genus level richness comparisons between the data sets obtained by the original and the modified primer pairs. The slope of the observed ASV and Shannon index were predicted by the linear regression analysis using the modified and original primer sets.

Table S9. Blast mapping of patescibacterial ASVs to Danish WWTP metagenomic datasets. Three 100% identity mapping ASVs with >330 base pair coverage were highlighted in yellow.

Table S10. Global distribution of the patescibacterial genera identified as core community members from each country (average relative abundance).

Table S11. Core patescibacterial genera identified if all four main process types of activated sludge samples were analysed together and MAG representation of core genera.

Table S12. Core patescibacterial genera identified in activated sludge samples from plants with carbon removal (C).

Table S13. Core patescibacterial genera identified in activated sludge samples from plants with carbon removal with nitrification (C, N).

Table S14. Core patescibacterial genera identified in activated sludge samples from plants with carbon removal, nitrification and denitrification (C, N, DN).

Table S15. Core patescibacterial genera identified in activated sludge samples from plants with carbon removal, nitrogen removal, enhanced biological phosphorus removal (C, N, DN, P / EBPR).

Table S16. List of patescibacterial genera that were characterized as CRAT genera

Table S17. Correlated genus pairs (X and Y) with p value <0.001 and an absolute correlation value >0.5 identified by SparCC network analysis.

Table S18. Correlated genus pairs (X and Y) with p value <0.001 and an absolute correlation value >0.5 identified by CoNet network analysis.

Table S19. Correlated genus pairs (X and Y) with an absolute weight edge >0.2 identified by SPIEC-EASI network analysis.

Table S20. Correlated ASV pairs (X and Y) with p value <0.001 and an absolute correlation value >0.5 identified by SparCC network analysis.

Table S21. Correlated ASV pairs (X and Y) with p value <0.001 and an absolute correlation value >0.5 identified by CoNet network analysis.

Table S22. Correlated ASV pairs (X and Y) with an absolute weight edge >0.2 identified by SPIEC-EASI network analysis.
